# Esketamine Enhances the Chemosensitivity of Colorectal Adenocarcinoma Cells to 5-Fluorouracil via AMPK/mTOR/HMMR Signaling Pathway

**DOI:** 10.32604/or.2025.072563

**Published:** 2026-01-19

**Authors:** Yuerou Feng, Panpan Tong, Shuwen Fu, Xiaofan Lu, Liquan Zheng, Jielan Lai, Renchun Lai

**Affiliations:** Department of Anesthesiology, State Key Laboratory of Oncology in South China, Guangdong Provincial Clinical Research Center for Cancer, Sun Yat-sen University Cancer Center, Guangzhou, 510060, China

**Keywords:** Esketamine, 5-fluorouracil, colorectal adenocarcinoma, chemosensitivity, AMPK/mTOR/HMMR pathway

## Abstract

**Background:**

The efficacy of standard 5-fluorouracil (5-FU) chemotherapy for colorectal cancer is limited by drug resistance and adverse effects, prompting research into esketamine, a potent ketamine variant with analgesic, antidepressant, and recently discovered anti-tumor properties, to determine if it can enhance 5-FU’s chemosensitivity. This study investigates whether esketamine synergizes with 5-FU to enhance therapeutic efficacy in colorectal adenocarcinoma cell models.

**Methods:**

We performed functional assays to evaluate proliferation (CCK-8), migration (wound healing), invasion (Transwell), and apoptosis (flow cytometry) in colorectal adenocarcinoma cell lines treated with 5-FU alone or in combination with esketamine. Transcriptomic profiling was conducted using RNA sequencing, and Kyoto Encyclopedia of Genes and Genomes (KEGG) pathway enrichment analysis was employed to identify critical molecular targets and signaling networks. Protein-level validation of key pathway components was performed via western blotting.

**Results:**

Combination therapy with esketamine and 5-FU synergistically inhibited cellular proliferation, migration, and invasion while significantly inducing apoptosis compared to monotherapy. Mechanistically, esketamine potentiated 5-FU-driven AMP-activated protein kinase (AMPK) phosphorylation, leading to inhibition of both mammalian target of rapamycin (mTOR) and hyaluronan-mediated motility receptor (HMMR).

**Conclusion:**

Esketamine enhances 5-FU chemosensitivity in colorectal adenocarcinoma by activating the AMPK/mTOR/HMMR signaling axis, thereby suppressing tumor progression and metastatic potential. These findings position esketamine as a potential adjunctive therapy for 5-FU-based regimens, offering the dual benefit of enhancing chemotherapeutic efficacy while addressing cancer-associated comorbidities including pain and depression.

## 1 Introduction

Colorectal cancer (CRC) remains a leading cause of gastrointestinal malignancy-related mortality worldwide [1,2], characterized by aggressive tumor proliferation and chemoresistance that pose significant challenges to effective clinical management. While 5-fluorouracil (5-FU) and platinum-based regimens constitute first-line therapies, tumor cells frequently develop acquired resistance through adaptive molecular mechanisms, ultimately limiting therapeutic outcomes [3]. There is thus an urgent need for combinatorial strategies that enhance 5-FU chemosensitivity while minimizing off-target toxicity.

Chronic pain and central sensitization are well-established complications in CRC survivors [4,5], significantly impairing both physical and psychological well-being [6]. The prevalence of anxiety and depression among CRC patients ranges from 1.0%–47.2% and 1.6%–57.0%, respectively [7]. Ketamine, a non-competitive N-methyl-D-aspartate (NMDA) receptor antagonist, exhibits dual therapeutic potential as both an analgesic for cancer-related neuropathic pain [8] and an antidepressant. A randomized controlled trial demonstrated that a single intravenous ketamine dose (0.5 mg/kg) alleviated treatment-resistant depressive symptoms within 110 min of administration [9]. Recent evidence suggests that ketamine influences cell proliferation and apoptosis in various cancer types, including pancreatic cancer [10], hepatocellular carcinoma [11], and lung adenocarcinoma [12]. Notably, ketamine inhibits the malignant potential of colorectal cancer cells by blocking NMDA receptor signaling [13]. Esketamine, the pharmacologically active S-enantiomer of ketamine, demonstrates approximately 2-fold greater NMDA receptor affinity and analgesic potency compared to racemic ketamine, while maintaining similar mechanistic profiles [14]. Current clinical research primarily focuses on its perioperative analgesic efficacy and utility in treatment-resistant depression [15–19]. However, its direct antitumor effects and potential synergism with chemotherapeutic agents remain largely unexplored. Crucially, no studies have investigated whether esketamine enhances 5-FU’s antitumor activity or influences disease progression when administered for pain/depression management in advanced CRC patients undergoing chemotherapy

To address these knowledge gaps, we evaluated esketamine’s effects on cellular activity and 5-FU chemosensitivity in human colorectal adenocarcinoma models (DLD1 and HCT15 cell lines). Therefore, this study aimed to investigate whether esketamine enhances the chemosensitivity of CRC cells to 5-FU and to examine the underlying hypothesis that this synergistic effect is mediated through the AMPK/mTOR/HMMR signaling axis.

## 2 Material and Methods

### 2.1 Cell Lines and Cell Culture

Human colorectal adenocarcinoma cell lines DLD1 (ATCC^®^ CCL-221™) and HCT15 (ATCC^®^ CCL-225™) were obtained from the American Type Culture Collection (ATCC, Manassas, VA, USA). All cell lines were authenticated using short tandem repeat (STR) profiling and routinely tested for mycoplasma contamination using the MycoAlert™ Mycoplasma Detection Kit (Lonza, Catalog No.: LT07-118) prior to experimental use. Cells were cultured in RPMI 1640 medium (Invitrogen, Carlsbad, CA, USA; Catalog No.: 21870084) supplemented with 10% fetal bovine serum (FBS; Gibco, Grand Island, NY, USA; Catalog No.: 12657029) and 1% penicillin-streptomycin solution (100 U/mL penicillin and 100 µg/mL streptomycin; Invitrogen; Catalog No.: 15140122). Cultures were maintained at 37°C in a humidified incubator with 5% CO_2_. Cells were passaged every 2–3 days at 80%–90% confluence using 0.25% trypsin-EDTA (Gibco; Catalog No.: 25300054) and used within 20 passages to ensure genetic stability.

### 2.2 Reagents and Antibodies

5-Fluorouracil (5-FU; Cat. No. HY-90006) was purchased from MedChemExpress (Monmouth Junction, NJ, USA) and dissolved in dimethyl sulfoxide (DMSO; Sigma-Aldrich, St. Louis, MO, USA, Catalog No.: D2650) to prepare a 100 mM stock solution, which was stored at −20°C. Working concentrations were prepared by diluting the stock in complete culture medium, ensuring that the final DMSO concentration did not exceed 0.1%. Esketamine (Batch No.: 211026BL) was obtained from Jiangsu Hengrui Medicine Co., Ltd. (Lianyungang, China).

The following primary and secondary antibodies were used for western blot analysis: anti-GAPDH (1:5000, Proteintech, 60004-1-Ig, Wuhan, China), anti-cleaved caspase-3 (1:1000, Proteintech, 68773-1-Ig), anti-caspase-3 (1:1000, Wanleibio, WL04004, Shenyang, China), anti-Bcl-2 (1:1000, Wanleibio, WL01556), anti-Bax (1:1000, Wanleibio, WL01637, anti-E-cadherin (1:1000, Santa Cruz Biotechnology, sc-21791, Dallas, TX, USA), anti-N-cadherin (1:1000, Proteintech, 66219-1-Ig, anti-Matrix metallopeptidase 9 (MMP-9, 1:1000, Santa Cruz Biotechnology, sc-393859), anti-AMPK (1:1000, Cell Signaling Technology, #2532, Danvers, MA, USA), anti-p-AMPK (Thr172) (1:1000, Cell Signaling Technology, #2531), anti-mTOR (1:1000, Cell Signaling Technology, #2983), anti-p-mTOR (Ser2448) (1:1000, Cell Signaling Technology, #5536), and anti-HMMR (1:500, Abcam, ab124729, Cambridge, UK). The HRP-conjugated secondary antibodies included goat anti-rabbit IgG (1:5000, Cell Signaling Technology, #7074) and goat anti-mouse IgG (1:5000, Cell Signaling Technology, #7076).

### 2.3 Cell Counting Kit-8 (CCK8) Assay

Cell viability was assessed using the CCK-8 assay (Dojindo Molecular Technologies, Kumamoto, Japan). Briefly, DLD1 and HCT15 cells were seeded in 96-well plates (Corning) at a density of 1 × 10^4^ cells per well in 100 µL of complete medium and allowed to adhere overnight. Cells were then treated with a range of esketamine concentrations (0–2 mM) and/or 5-FU (0–150 µM) for 48 h. Six replicate wells were used per condition. After treatment, 10 µL of CCK-8 reagent was added to each well, and plates were incubated for 4 h at 37°C. Absorbance was measured at 450 nm using a BioTek Synergy H1 microplate reader (Agilent Technologies, Santa Clara, CA, USA).

### 2.4 Synergistic Interaction Analysis

The combined effect of 5-FU and esketamine was evaluated using the Chou-Talalay method via CompuSyn software (ComboSyn, Inc., Paramus, NJ, USA). DLD1 and HCT15 cells were treated with serial dilutions of 5-FU and esketamine alone or in combination at fixed molar ratios for 48 h. The 5-FU concentration ranged from 0 to 150 μM, while esketamine was tested from 0.5 to 1.5 mM. The combination index (CI) values were calculated based on the median-effect principle using data from the CCK-8 assay, with CI <1, =1, or >1 indicating synergy, additivity, or antagonism, respectively [20].

### 2.5 Colony Formation Assay

DLD1 and HCT15 cells were seeded in 6-well plates (Corning) at a density of 1 × 10^3^ cells per well and allowed to grow for 14 days with medium refreshed every 3 days. Colonies were fixed with 4% paraformaldehyde (Beyotime, Catalog No.: P0099, Shanghai, China) for 20 min, stained with 0.5% crystal violet (Sigma-Aldrich) for 30 min, and washed gently with PBS. Plates were air-dried, and colonies containing >50 cells were counted manually.

### 2.6 EdU Staining Assay

Cell proliferation of DLD1 and HCT15 cells was assessed using the BeyoClick™ EdU Kit (Beyotime C0078S). Cells were incubated with 10 µM EdU for 4 h at 37°C, fixed with 4% paraformaldehyde, permeabilized with 0.3% Triton X-100, and incubated with Click Reaction Buffer for 30 min in the dark. Nuclei were counterstained with Hoechst 33342. Images were acquired using a Leica TCS SP8 confocal microscope (Leica Microsystems, Wetzlar, Germany), and EdU-positive cells were quantified using ImageJ software (version 1.54r, National Institutes of Health, Bethesda, MD, USA).

### 2.7 Wound Healing Assays

For the wound healing assay, cells were seeded at a high density (2 × 10^5^ cells per well) in 6-well plates and incubated until they reached 95%–100% confluence. A uniform scratch wound was then introduced into the monolayer using a sterile 200 µL pipette tip. After washing with PBS to remove debris, cells were treated with esketamine, 5-FU, or their combination in serum-free medium. Wound images were captured at 0 and 48 h using a Nikon Eclipse Ti2 microscope (Nikon Corporation, Tokyo, Japan). Migration distance was measured using ImageJ software (version 1.54r, National Institutes of Health), and wound closure percentage was calculated.

### 2.8 Transwell Assay

Transwell inserts (8 µm pore size, Corning, Catalog No.: 3428) were coated with Matrigel Matrix (Corning, Catalog No.: CB40234C) diluted to a working concentration of 1–2 mg/mL in pre-cooled serum-free medium and allowed to solidify for 1 h at 37°C. A total of 5 × 10^4^ DLD1 or HCT15 cells in 200 µL of serum-free RPMI 1640 medium were seeded into the upper chamber. The lower chamber was filled with 600 µL of complete RPMI 1640 medium supplemented with 10% FBS to serve as a chemoattractant. After 48 h, non-invaded cells were removed from the upper surface, and invaded cells on the lower membrane were fixed, stained with 1% crystal violet, and imaged using a Motic BA310 microscope (Motic, Xiamen, China). Cells were counted in three random fields per insert.

### 2.9 Apoptosis Assay

Apoptosis was assessed using an Annexin V-FITC Apoptosis Detection Kit (BD Biosciences, Catalog No.: 556547, San Jose, CA, USA). After a 48-h treatment with esketamine and/or 5-FU, DLD-1 and HCT-15 cells were harvested, washed with PBS, and resuspended in binding buffer. A total of 1 × 10^5^ cells were resuspended in 100 µL of binding buffer and stained with 5 µL of Annexin V-FITC and 5 µL of PI for 15 min in the dark. Cells were analyzed immediately using a CytoFLEX S flow cytometer (Beckman Coulter, Inc., United States). Data were analyzed using CytExpert software (version 2.3; Beckman Coulter, Inc., United States).

### 2.10 RNA Extraction and Sequencing

Total RNA was extracted from cells using TRIzol^®^ reagent (Invitrogen, Thermo Fisher Scientific, Inc., Waltham, MA, USA, Catalog No.: 15596026) following the manufacturer’s instructions. RNA concentration and purity were assessed using a NanoDrop ND-1000 spectrophotometer (NanoDrop Technologies; Thermo Fisher Scientific, Inc., Wilmington, DE, USA), and RNA integrity was verified by normal denaturing agarose gel electrophoresis. The RNA Clean XP Kit (Beckman Coulter, Inc., Brea, CA, USA, Catalog No.: A63987) was used for further purification.

RNA-sequencing (RNA-seq) libraries were prepared from poly(A)+ enriched RNA using a standard dUTP-based strand-specific method and sequenced on an Illumina HiSeq 2000/2500 platform (Illumina, Inc., San Diego, CA, USA) [21]. The resulting sequencing data exhibited high quality, with a sequencing depth of approximately 6 G bases per sample and Q30 scores above 85% for all samples. All libraries were processed in a single sequencing batch to minimize technical batch effects; therefore, no batch-effect correction was applied during subsequent bioinformatic analysis.

For data processing, the Seurat R package (version 4.3.0) was utilized to import the raw data and perform quality control, normalization, scaling, and downstream analyses. Low-quality cells were filtered out using strict thresholds: cells with fewer than 501 expressed genes or 1001 UMIs, as well as those with mitochondrial gene counts exceeding 25%, were discarded. The remaining high-quality cells were normalized and scaled using default parameters. Highly variable features were identified using the ‘FindVariableFeatures’ function. Principal Component Analysis (PCA) was performed on the scaled data based on these variable features. Dimensional reduction and cell clustering were then carried out using the ‘FindNeighbors’ (dims = 1:10) and ‘FindClusters’ (resolution = 0.5) functions, respectively. Nonlinear dimensional reduction techniques (t-SNE and UMAP) were subsequently applied for data visualization and exploration. Differentially expressed long non-coding RNAs and mRNAs were identified using the DESeq2 package (version 1.46.0) with the following thresholds: adjusted *p*-value (FDR) < 0.01 and absolute fold-change > 2.

### 2.11 Kyoto Encyclopedia of Genes and Genomes (KEGG) Pathway Annotation

The KEGG pathway database (https://www.kegg.jp/kegg/pathway.html, accessed on 13 December 2021) was used to identify signaling pathways associated with differentially expressed mRNAs. KEGG pathway enrichment analysis was conducted using the clusterProfiler R package (version 4.14.3, Bioconductor, Seattle, WA, USA) [22]. A *p*-value of less than 0.05 was considered to indicate a statistically significant correlation between the mRNAs and their associated functions and/or pathways.

### 2.12 Western Blot Analysis

The protein concentration was determined by the Bradford method. Protein lysates (20 μg per sample) were resolved by electrophoresis on 12.5% SDS-polyacrylamide gels and transferred to polyvinylidene fluoride (PVDF) membranes (MilliporeSigma, Catalog No.: IPVH00010, Burlington, MA, USA). Membranes were blocked with 5% bovine serum albumin (BSA; Sigma-Aldrich, Catalog No.: A7030, St. Louis, MO, USA) in Tris-buffered saline containing 0.1% Tween-20 (TBST) and then incubated overnight at 4°C with the following primary antibodies. After washing, membranes were incubated with horseradish peroxidase (HRP)-conjugated secondary antibodies for 1 h at room temperature. BeyoECL Plus chemiluminescent substrate (Beyotime, Catalog No.: P0018S) was used for signal detection. Band intensities were quantified using ImageJ software (version 1.8.0, National Institutes of Health).

### 2.13 Overexpression of HMMR

To establish stable HMMR-overexpressing cell lines, the full-length coding sequence of human HMMR was cloned into the lentiviral expression vector pLVX-puro (Clontech Laboratories, Inc., Mountain View, CA, USA). The recombinant plasmid and the packaging plasmids (psPAX2 and pMD2.G) were co-transfected into HEK-293 cells (ATCC^®^ CRL-3216™) at 60%–70% confluence using Lipofectamine 3000 (Invitrogen) according to the manufacturer’s protocol. The lentivirus-containing supernatant was collected 48 and 72 h post-transfection, filtered through a 0.45-μm PVDF filter (MilliporeSigma), and concentrated by ultracentrifugation at 76,900× *g* for 18 h at 4°C.

DLD1 and HCT15 cells were then transduced with the harvested lentivirus in the presence of 8 μg/mL Polybrene (Sigma-Aldrich). After 24 h, the virus-containing medium was replaced with fresh complete medium. To select stably transduced cells, puromycin dihydrochloride (AbMole, Catalog No.: M3637) was added to the culture medium at a final concentration of 2 μg/mL for DLD1 and 1.5 μg/mL for HCT15 for one week. The overexpression efficiency of HMMR was confirmed by western blot analysis as described in Section 2.12.

### 2.14 Statistical Analysis

The data are presented as the mean ± standard deviation (SD) from at least three independent biological replicates. The normality of data distribution was assessed using the Shapiro-Wilk test, and the homogeneity of variances was verified using Levene’s test. For comparisons between two groups, an unpaired Student’s *t*-test was employed. Comparisons among multiple groups were performed using one-way analysis of variance (ANOVA), followed by Tukey’s post hoc test for data meeting assumptions of normality and homogeneity of variances. For data that did not meet these assumptions, the Kruskal-Wallis test was used, followed by Dunn’s post hoc test. All statistical analyses were conducted using GraphPad Prism version 9.0 (GraphPad Software, Inc., San Diego, CA, USA). Dose-response curves were generated, and IC_50_ values were calculated using nonlinear regression in GraphPad Prism 9.0. A *p*-value of less than 0.05 (*p* < 0.05) was considered statistically significant.

## 3 Results

### 3.1 Effect of Esketamine and 5-FU Alone or in Combination on Cell Viability

**Figure 1:**
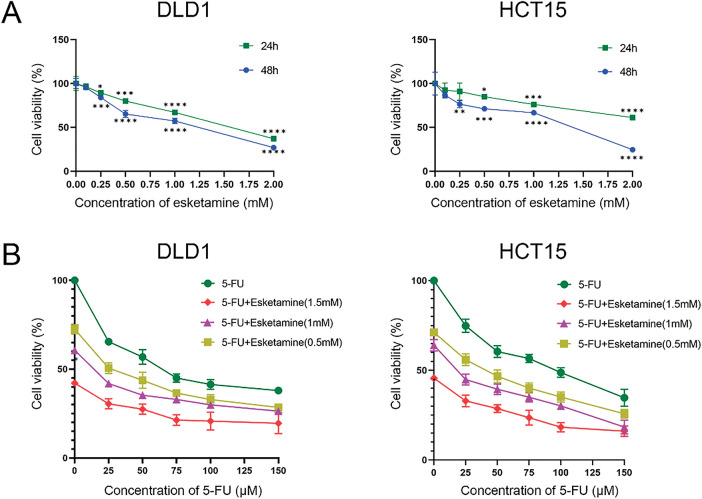

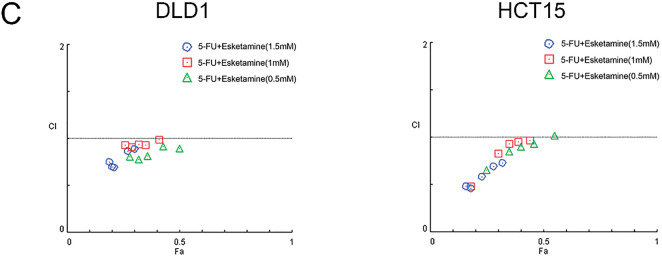
**Esketamine synergizes with 5-FU to inhibit colon cancer cell viability.** (**A**) DLD1 and HCT15 cells were treated with the indicated concentrations of esketamine for 24 h or 48 h. (**B**) Cells were treated with the indicated concentrations of 5-FU alone or in combination with esketamine for 48 h. Cell viability was measured by Cell counting Kit-8 (CCK-8) assay. Data are normalized to the vehicle control (DMSO) group and presented as the mean ± SD (*n* = 3 independent experiments). *p < 0.05, **p < 0.01, ***p < 0.001, ****p < 0.0001 vs. control group (unless otherwise specified). (**C**) CI values were calculated using the Chou-Talalay method via CompuSyn software after 48 h of combination treatment. CI < 1 indicates synergy. 5-FU, 5-fluorouracil. CI, combination index

To determine whether esketamine enhances the chemotherapeutic efficacy of 5-FU, we assessed its impact on cell viability using the CCK-8 assay. As shown in Fig. 1A, treatment with esketamine (0–2.0 mM) for 24 or 48 h dose-dependently reduced the viability of DLD1 and HCT15 cells. Similarly, 5-FU (0–150 μM) also suppressed cell viability in a dose-dependent manner (Fig. 1B). More importantly, the combination of esketamine (0.5–1.5 mM) and 5-FU (0–150 μM) for 48 h significantly enhanced the suppression of cell viability compared to 5-FU alone (Fig. 1B).

To quantitatively evaluate the pharmacodynamic interaction between 5-FU and esketamine, we applied the Chou-Talalay method and calculated the combination index (CI) using CompuSyn software. The Fa-CI plots demonstrated that most CI values were below 1 across a wide range of effect levels (Fa) in both cell lines (Fig. 1C), indicating a synergistic interaction between the two drugs. This synergistic effect was further visualized in a dose-effect matrix heatmap (Supplementary Fig. S1).

### 3.2 Esketamine and 5-FU Synergistically Inhibit Cell Proliferation

**Figure 2:**
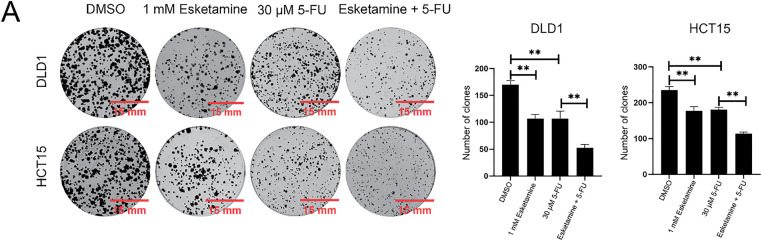

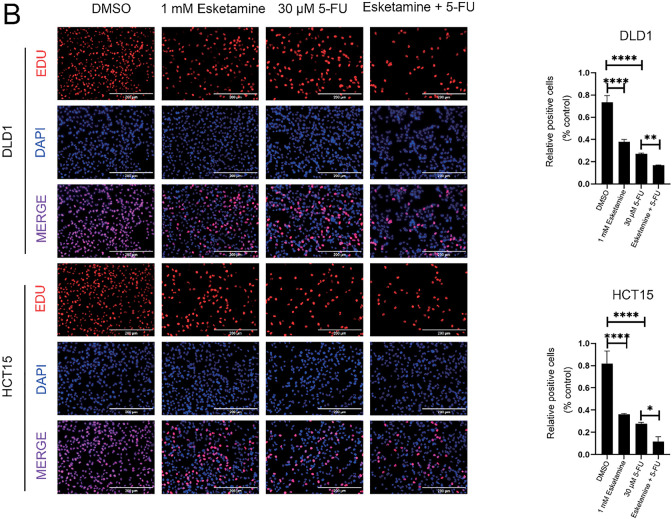
**Esketamine enhances the anti-proliferative effect of 5-FU in colon cancer cells.** (**A**) Representative images (left) and quantification (right) of colony formation assays in DLD1 and HCT15 cells treated with esketamine (1 mM), 5-FU (30 µM), or their combination. Scale bars, 15 mm. (**B**) Representative images (left) and quantification (right) of EdU incorporation assays. Scale bars, 200 µm. For all panels, data are presented as the mean ± SD (*n* = 3 independent experiments). *p < 0.05, **p < 0.01, ****p < 0.0001

As uncontrolled proliferation is a hallmark of colon cancer progression, we next evaluated the effect of combined esketamine (1 mM) and 5-FU (30 μM) treatment on colorectal adenocarcinoma cell proliferation. Colony formation and EdU assays revealed that the combination treatment significantly enhanced the suppression of proliferation compared to either agent alone in both DLD1 and HCT15 cells (Fig. 2). These results indicate that concurrent administration of esketamine and 5-FU enhances the anticancer efficacy of 5-FU in colon cancer.

### 3.3 Combined Esketamine and 5-FU Treatment Suppresses Cell Migration and Invasion

Epithelial–mesenchymal transition (EMT) is closely associated with the pathogenesis of colon cancer and plays a critical role in driving invasion and metastasis [23]. During EMT, cell migratory and invasive capacities are markedly enhanced [24,25]. We therefore investigated whether esketamine augments the inhibitory effect of 5-FU on colon cancer cell migration and invasion.

**Figure 3:**
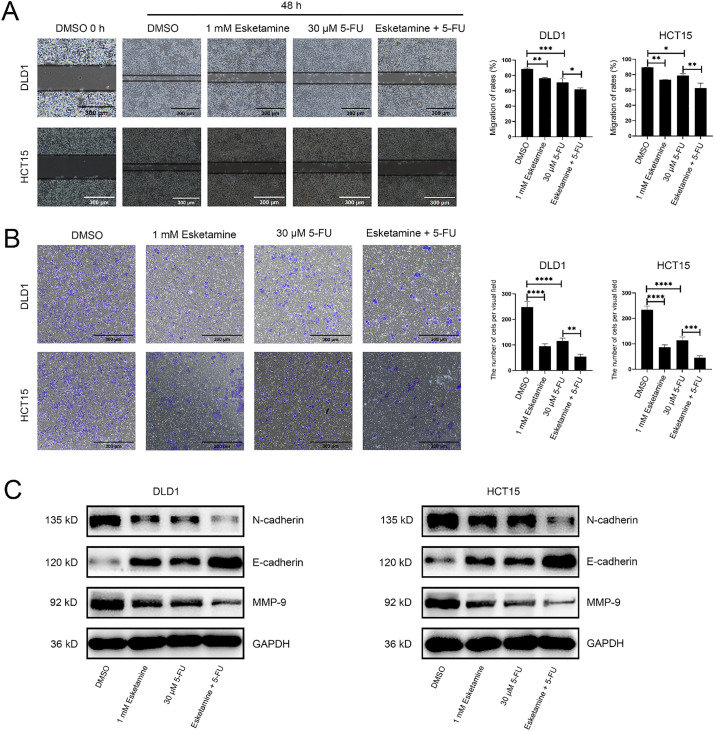
**The combination of esketamine and 5-FU suppresses migration, invasion, and modulates associated protein expression.** (**A**) Wound healing assays showing representative images (left) and quantified migration rates (right) of DLD1 and HCT15 cells after 48 h of treatment with esketamine (1 mM), 5-FU (30 µM), or their combination. Scale bars, 300 µm. (**B**) Transwell invasion assays showing representative images (left) and quantification of invaded cells (right). Scale bars, 300 µm. (**C**) Western blot analysis of E-cadherin, N-cadherin, and Matrix metallopeptidase 9 (MMP-9) protein levels in cells treated for 48 h. GAPDH served as the loading control. Data are presented as the mean ± SD (*n* = 3 independent experiments). *p < 0.05, **p < 0.01, ***p < 0.001, ****p < 0.0001

Wound healing assays showed that treatment with 5-FU (30 μM) or esketamine (1 mM) alone suppressed cell migration, whereas the combination of both agents resulted in a substantially greater inhibition (Fig. 3A). In control groups, the wound was largely closed by migrating cells within 48 h, whereas cells treated with the drug combination failed to close the wound. Quantitative analysis confirmed that the combination treatment more effectively impeded cell migration than either single agent (Fig. 3A).

We further evaluated the effect on invasive ability using a Transwell invasion assay. As shown in Fig. 3B, 5-FU significantly reduced the number of invading cells compared to the control. The combination treatment further suppressed Matrigel penetration. Quantitative analysis demonstrated that the combination therapy more potently inhibited cell invasion compared to 5-FU or esketamine alone (Fig. 3B).

We also examined the expression of key proteins associated with cell migration and invasion. MMP-9, a molecule critically involved in cell migration, was downregulated in DLD1 and HCT15 cells following combination treatment (Fig. 3C). In addition, the combination treatment increased the expression of the epithelial marker E-cadherin and decreased that of the mesenchymal marker N-cadherin (Fig. 3C), consistent with the suppression of EMT [26]. Together, these findings confirm that esketamine enhances the 5-FU–induced inhibition of migration and invasion in colon cancer cells.

### 3.4 Combined Esketamine and 5-FU Treatment Promotes Apoptosis

**Figure 4:**
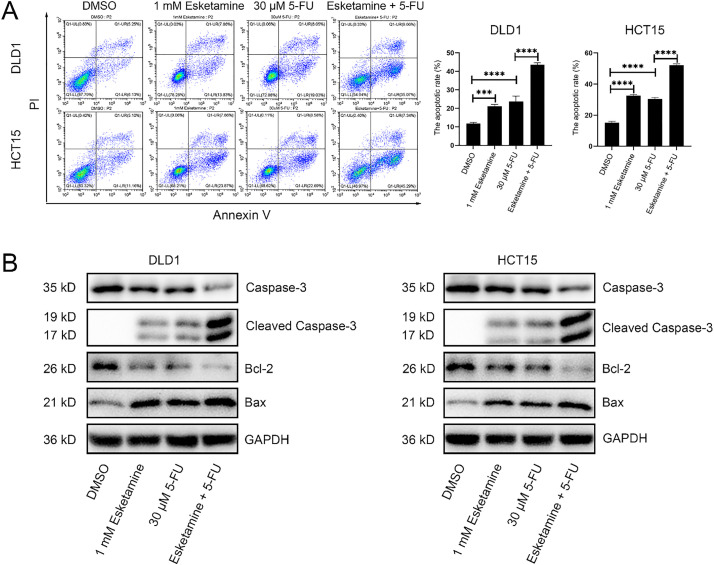
**Co-treatment with esketamine and 5-FU promotes apoptosis in colon cancer cells.** (**A**) Flow cytometry analysis of Annexin V/PI staining in DLD1 and HCT15 cells treated with esketamine (1 mM), 5-FU (30 µM), or their combination for 48 h. The quantifications of apoptotic cells are shown. (**B**) Western blot analysis of the indicated apoptosis-related proteins. GAPDH served as the loading control. Data are presented as the mean ± SD (*n* = 3 independent experiments). ***p < 0.001, ****p < 0.0001

We used flow cytometry to analyze the effect of esketamine and 5-FU on apoptosis. Further quantification of apoptosis stages revealed that the combination of esketamine (1 mM) and 5-FU (30 μM) significantly increased early apoptosis in both cell lines. Although a significant increase in late apoptosis was observed in DLD1 cells, the increase in HCT15 cells did not reach statistical significance compared to 5-FU monotherapy. Nevertheless, the total apoptosis rate (early + late) was significantly elevated by the combination treatment in both DLD1 and HCT15 cells (Figs. 4A and S2).

Western blot analysis showed that the combination treatment altered the levels of key apoptosis-related proteins—caspase-3, cleaved caspase-3, Bcl-2, and Bax—in both cell lines after 48 h. Specifically, the combination treatment upregulated cleaved caspase-3 and Bax, and downregulated caspase-3 and Bcl-2 (Fig. 4B).

### 3.5 Global Gene Expression and Fold-Change (FC) Analysis by RNA-Seq

**Figure 5:**
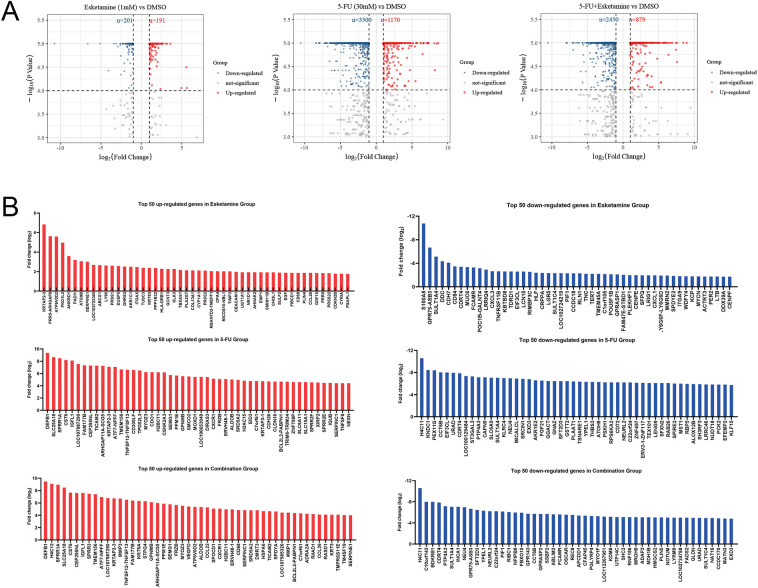

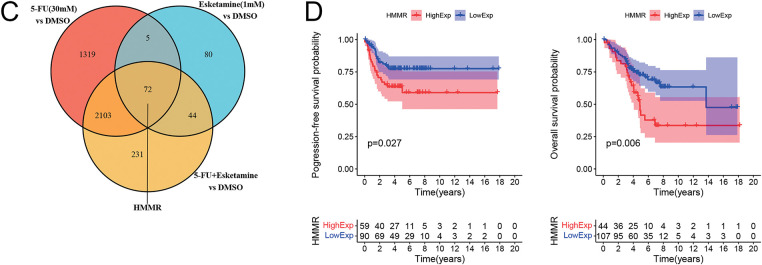
**Transcriptomic analysis identifies HMMR as a key downregulated gene associated with poor prognosis.** (**A**) Volcano plots showing differentially expressed genes (DEGs) in DLD1 cells treated with esketamine (1 mM), 5-FU (30 µM), or their combination for 48 h compared to the DMSO control. Red and blue dots represent significantly upregulated and downregulated genes, respectively (|log_2_FC|> 1, FDR < 0.05). (**B**) Bar graphs displaying the top 50 upregulated and downregulated genes in each treatment group. (**C**) Venn diagram illustrating the overlap of downregulated DEGs among the three treatment groups, highlighting HMMR as a common target. (**D**) Kaplan-Meier survival analysis from the GEO dataset (GSE103479) showing the correlation between high HMMR expression and poor progression-free survival (PFS) and overall survival (OS) in CRC patients. FDR, false discovery rate; FC, fold change

We performed RNA-seq on DLD1 cells to explore the transcriptional mechanisms underlying the anti-CRC effects of esketamine, 5-FU, and their combination. Differentially expressed genes (DEGs) were identified using the thresholds |log_2_FC| > 1 and FDR < 0.05. We identified 392 DEGs in the esketamine group (191 up, 201 down), 4670 in the 5-FU group (1170 up, 3500 down), and 3329 in the combination group (879 up, 2450 down) (Fig. 5A). The top 50 up- and downregulated genes in each group are displayed in Fig. 5B.

Venn diagram analysis revealed 72 downregulated DEGs common to all three treatment groups (Fig. 5C). Cross-referencing with published literature highlighted HMMR as a gene implicated in CRC pathogenesis [27–29]. To assess the clinical relevance of HMMR, we analyzed its correlation with patient prognosis using the GEO dataset (GSE103479). Kaplan–Meier analysis showed that high HMMR expression was associated with significantly shorter overall survival (OS) and progression-free survival (PFS) (Fig. 5D), supporting its role as a critical oncogenic driver in CRC.

### 3.6 Signaling Pathways Modulated by Esketamine and 5-FU Alone or in Combination

**Figure 6:**
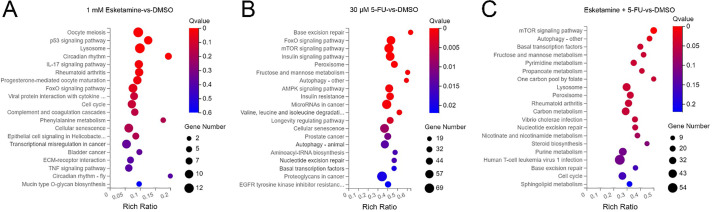
**Kyoto Encyclopedia of Genes and Genomes (KEGG) pathway enrichment analysis of differentially expressed genes.** Bubble charts showing the significantly enriched KEGG pathways for DEGs in DLD1 cells from the (**A**) esketamine, (**B**) 5-FU, and (**C**) combination treatment groups. The Q-value is used to evaluate the enrichment significance

KEGG pathway enrichment analysis was performed to identify signaling pathways affected by esketamine and 5-FU. In the esketamine group, DEGs were enriched in cancer-related pathways such as “p53 signaling,” “IL-17 signaling,” “FoxO signaling,” “Cell cycle,” “TNF signaling,” “Bladder cancer,” and “Transcriptional misregulation in cancer” (Fig. 6A). In the 5-FU group, enriched terms included “FoxO signaling,” “AMPK signaling,” “mTOR signaling,” “Autophagy,” “EGFR tyrosine kinase inhibitor resistance,” “Proteoglycans in cancer,” “Prostate cancer,” and “MicroRNAs in cancer” (Fig. 6B). The combination group showed notable enrichment in “mTOR signaling,” “Autophagy,” and “Cell cycle” pathways (Fig. 6C).

### 3.7 Combined Esketamine and 5-FU Treatment Regulates the AMPK/mTOR/HMMR Axis in Human Colon Cancer

**Figure 7:**
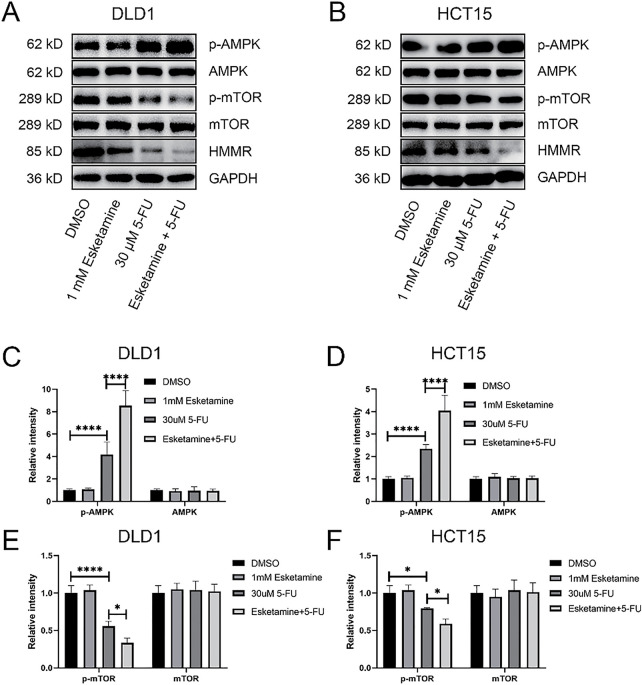

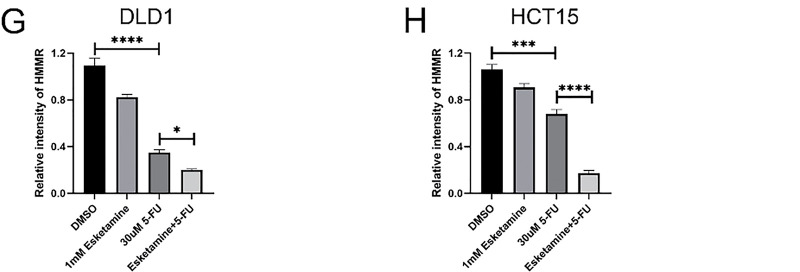
**The combination therapy activates the AMPK/mTOR/HMMR signaling axis.** (**A**) Western blot analysis of key proteins in the AMPK/mTOR/HMMR pathway in DLD1 cells treated with 5-FU (30 µM), esketamine (1 mM), or their combination for 48 h. (**B**) Western blot analysis of the same pathway proteins in HCT15 cells under identical treatment conditions. GAPDH served as the loading control for both cell lines. (**C**,**D**) Densitometric quantification of p-AMPK and AMPK protein levels in (**C**) DLD1 and (**D**) HCT15 cells. (**E**,**F**) Densitometric quantification of p-mTOR and mTOR protein levels in (**E**) DLD1 and (**F**) HCT15 cells. (**G**,**H**) Densitometric quantification of HMMR protein levels in (**G**) DLD1 and (**H**) HCT15 cells. Data are presented as the mean ± SD (*n* = 3 independent experiments). *p < 0.05, ***p < 0.001, ****p < 0.0001. p-, phosphorylated

Based on the KEGG results, which indicated enrichment of “p53 signaling” in the esketamine group, and “mTOR signaling,” “Autophagy,” and “AMPK signaling” in the 5-FU and combination groups, we further investigated whether the drug combination modulates the AMPK/mTOR/HMMR signaling axis. Western blot analysis showed that, compared to 5-FU alone, the combination treatment enhanced AMPK phosphorylation and suppressed the levels of phosphorylated mTOR and HMMR in both DLD1 and HCT15 cells (Fig. 7A,B). These results suggest that the combination inhibits mTOR signaling and downregulates HMMR via AMPK activation, contributing to its antitumor effects. Quantitative data are summarized in Fig. 7C–H.

To directly assess the functional role of HMMR, we established stable HMMR-overexpressing (HMMR-OE) DLD1 and HCT15 cell lines. As shown in Supplementary Fig. S3, HMMR overexpression significantly attenuated the inhibitory effect of the esketamine (1 mM) and 5-FU (30 μM) combination on cell viability in the CCK-8 assay. These results confirm that HMMR is a key functional mediator of the synergistic antitumor effect of esketamine and 5-FU co-treatment.

## 4 Discussion

While ketamine has been demonstrated to reduce CRC aggressiveness through NMDA receptor antagonism [13], the effects of its stereoselective S-enantiomer, esketamine—which exhibits enhanced pharmacodynamic activity—on CRC cells remain less thoroughly investigated. Our results establish that esketamine monotherapy suppresses proliferation, migration, and invasion, while promoting apoptosis in colorectal adenocarcinoma models (DLD1 and HCT15). These findings support the potential use of esketamine as a perioperative anesthetic in resectable colorectal adenocarcinoma, offering combined antitumor and analgesic benefits.

5-Fluorouracil (5-FU) remains a cornerstone of CRC chemotherapy, despite limitations such as dose-related toxicities and acquired resistance [30–34]. Previous studies have explored combinatorial approaches using natural compounds—such as puerarin [35], 6-gingerol [36], and melatonin [26]—to improve 5-FU efficacy. Although the role of 5-FU in CRC treatment is well established, and our study provides evidence of esketamine’s antitumor properties, no prior preclinical studies have systematically examined the combined effect of esketamine and 5-FU on colorectal cancer progression. Here, we show that co-treatment with esketamine and 5-FU significantly suppressed proliferation, migration, and invasion, and synergistically induced apoptosis in DLD1 and HCT15 human colorectal cancer cells. Analysis using the Chou–Talalay method yielded combination index (CI) values below 1.0 across multiple dose combinations, confirming pharmacodynamic synergy between the two agents. According to ATCC records, both DLD1 and HCT15 cell lines were derived from the same patient with colorectal adenocarcinoma. Thus, the esketamine–5-FU combination may represent a viable therapeutic strategy for patients with this malignancy.

Cell migration and invasion are critical drivers of colorectal cancer progression. In this study, combined treatment with esketamine and 5-FU synergistically reduced the migratory and invasive capabilities of DLD1 and HCT15 cells. This suppression likely results from the dual action of the drug combination: reducing cell viability while promoting apoptosis, thereby decreasing the pool of cells capable of migration and invasion. Furthermore, Western blot analysis revealed marked alterations in epithelial–mesenchymal transition (EMT) markers and extracellular matrix remodeling proteins, including downregulation of N-cadherin and MMP-9, and upregulation of E-cadherin. These molecular changes provide a mechanistic basis for the observed inhibition of tumor cell motility. Together, these results strongly support the conclusion that esketamine and 5-FU together exert potent inhibitory effects on colorectal cancer cell migration and invasion.

Apoptosis, a form of programmed cell death, represents a key mechanism by which cytotoxic agents eliminate cancer cells. Caspase-3, the main executioner caspase, plays a central role in mediating apoptotic signaling. Its activity is tightly regulated through complex molecular interactions under both physiological and pathological conditions [37]. The BCL-2 protein family, which includes both pro-apoptotic and anti-apoptotic members, forms the core regulatory network controlling mitochondrial-mediated apoptosis [38]. Among these, BAX serves as a critical pro-apoptotic effector that is maintained in an inactive state through interaction with anti-apoptotic proteins such as BCL-2 [39]. Our quantitative analysis revealed a marked increase in cleaved caspase-3 and BAX levels, accompanied by a pronounced decrease in BCL-2 and caspase-3 in the combination treatment group. These findings suggest that esketamine enhances the ability of 5-FU to induce apoptosis. Beyond apoptosis, our transcriptomic analysis suggests the potential involvement of additional cell death mechanisms. KEGG pathway enrichment analysis revealed that the combination treatment significantly altered genes involved in ‘Autophagy’ and ‘Cell cycle’ pathways (Fig. 6C), indicating that the therapeutic efficacy may extend beyond apoptotic cell death. The observed suppression of mTOR signaling (Fig. 7), a known regulator of both autophagy and cell cycle progression, provides a plausible mechanistic basis for these findings. While the current study focused primarily on apoptosis, future investigations will specifically examine the contribution of autophagy and cell cycle arrest to the overall antitumor effects.

The AMPK signaling pathway plays a critical role in regulating proliferation, migration, apoptosis, and autophagy in various cancer types [40–43]. AMPK activation has been shown to increase caspase-3 expression, leading to apoptosis in colon cancer cells [44]. In a colorectal adenoma model, AMPK activation promoted autophagy by upregulating Beclin-1 and LC3-II while downregulating phosphorylated mTOR [45]. The mTOR signaling pathway is critically involved in the progression of colorectal cancer (CRC), where it regulates key processes such as cell proliferation and migration [46]. This oncogenic pathway also contributes to the development and therapeutic resistance of other malignancies, including breast cancer, kidney cancer and glioblastoma [47–49]. In mammalian systems, AMPK can negatively regulate mTOR, thereby inducing apoptosis and autophagy in colon cancer both *in vitro* and *in vivo* [50]. Moreover, several studies have highlighted the role of HMMR in CRC. Sun et al. analyzed data from The Cancer Genome Atlas (TCGA) and Gene Expression Omnibus (GEO) and found that HMMR expression was significantly associated with overall survival in CRC patients [27]. HMMR has also been implicated in metastasis and treatment response in CRC [28,29]. Our analysis of the GEO dataset (GSE103479) corroborates these findings, showing that high HMMR expression correlates with poor patient outcomes. Western blot analysis revealed more pronounced changes in protein expression following combination treatment compared to monotherapy. Specifically, the combination group showed increased AMPK phosphorylation alongside reduced phosphorylation of mTOR and decreased HMMR levels. These results indicate that esketamine enhances the efficacy of 5-FU in colon cancer cells by promoting AMPK signaling and inhibiting mTOR and HMMR. Of note, esketamine alone did not affect the AMPK/mTOR pathway, suggesting that its mechanism of action in colon cancer cells requires further investigation.

To our knowledge, this study provides the first preclinical evidence for the chemopotentiating effect of esketamine on 5-FU in colorectal adenocarcinoma models. Our findings identify esketamine as a rational perioperative adjunct, with the dual potential to enhance chemotherapy efficacy while alleviating cancer-related pain and depression. Our study reveals esketamine’s intrinsic antitumor activity, as demonstrated by its dose-dependent inhibition of colorectal cancer cell proliferation and induction of apoptosis *in vitro*. This novel finding extends its potential utility beyond an analgesic adjunct. Therefore, esketamine represents a logical first-choice anesthetic for perioperative management in patients with operable colorectal adenocarcinoma.

However, several limitations should be noted: (1) *in vitro* models lack the complexity of the tumor microenvironment; (2) the high concentrations of esketamine required for efficacy *in vitro* may not be clinically achievable, and future studies should evaluate its selective cytotoxicity in normal colon epithelial cells to establish a therapeutic window; the clinical translatability thus requires further validation through pharmacokinetic/pharmacodynamic (PK/PD) modeling and *in vivo* studies; (3) the specificity of the AMPK/mTOR/HMMR pathway in response to esketamine merits further investigation; and (4) while our transcriptomic data suggest the involvement of additional cell death pathways such as autophagy and cell cycle arrest, direct experimental validation of these mechanisms was beyond the scope of this study. Future work should prioritize orthotopic CRC models, detailed cell cycle analysis, investigation of non-apoptotic cell death pathways, and biomarker-driven clinical trials to comprehensively evaluate the efficacy of this combination therapy.

## 5 Conclusion

Our findings demonstrate that esketamine synergizes with 5-FU to suppress colorectal adenocarcinoma progression through activation of caspase-3-mediated apoptosis and modulation of the AMPK/mTOR/HMMR signaling axis. These results position esketamine as a promising adjunct to 5-FU-based regimens, offering a dual therapeutic advantage by enhancing chemotherapeutic efficacy while potentially addressing cancer-related pain and depression. Further validation in preclinical models and biomarker-driven clinical trials is warranted to translate this combinatorial strategy into clinically actionable therapeutic paradigms.

## Supplementary Materials



## Data Availability

The data that support the findings of this study are available from the Corresponding Author, [Renchun Lai (RCL)], upon reasonable request.

## Supplementary Materials

The supplementary material is available online at https://www.techscience.com/doi/10.32604/or.2025.072563/s1.
