# Supplementary material for: Esketamine Enhances the Chemosensitivity of Colorectal Adenocarcinoma Cells to 5-Fluorouracil via AMPK/mTOR/HMMR Signaling Pathway
Source: Oncol Res. 2026 Jan 19;34(2):22. doi: 10.32604/or.2025.072563 (PMC12848708; doi:10.32604/or.2025.072563)
Supplement: Supplementary file 1 [file OncolRes-34-72563-s001.docx]

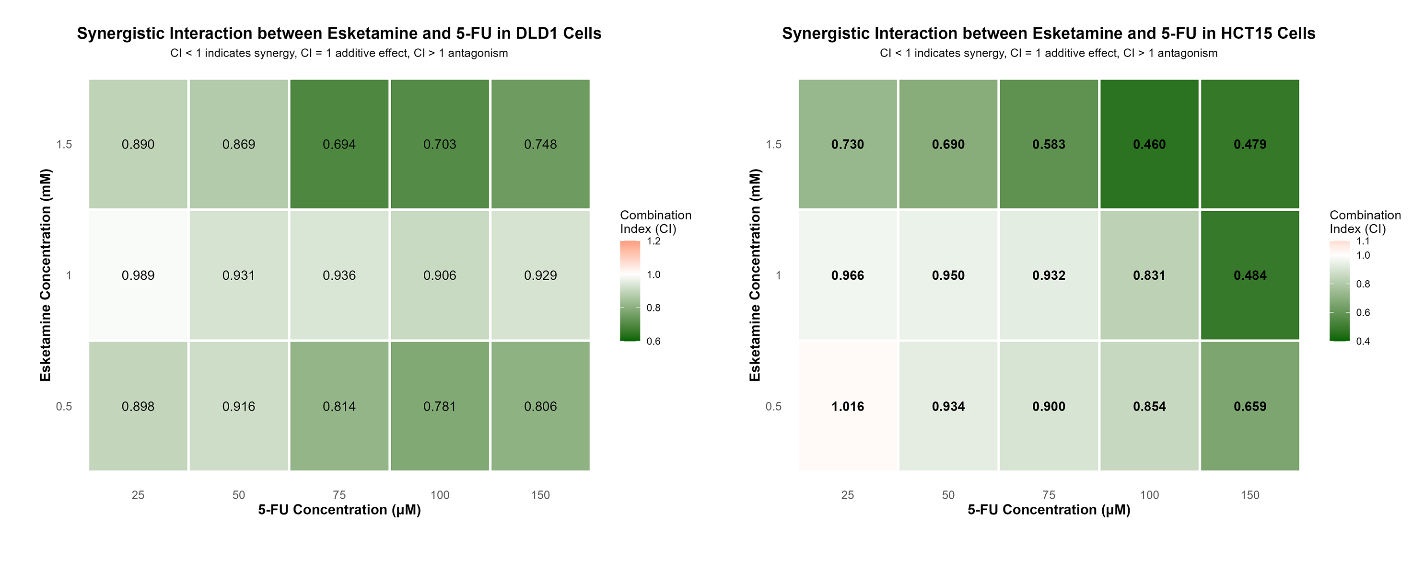


**Supplementary Figure S1. Dose-effect matrix analysis of the esketamine and 5-fluorouracil (5-FU) combination.** Heatmap depicting Combination Index (CI) values across a range of esketamine (0–1.5 mM) and 5-FU (0–150 μM) concentrations in DLD1 and HCT15 cells after 48 h of treatment. CI values were calculated using the Chou–Talalay method via CompuSyn software. CI < 1 (green) indicates synergy, CI = 1 (white) indicates additive effects, and CI > 1 (red) indicates antagonism.

**
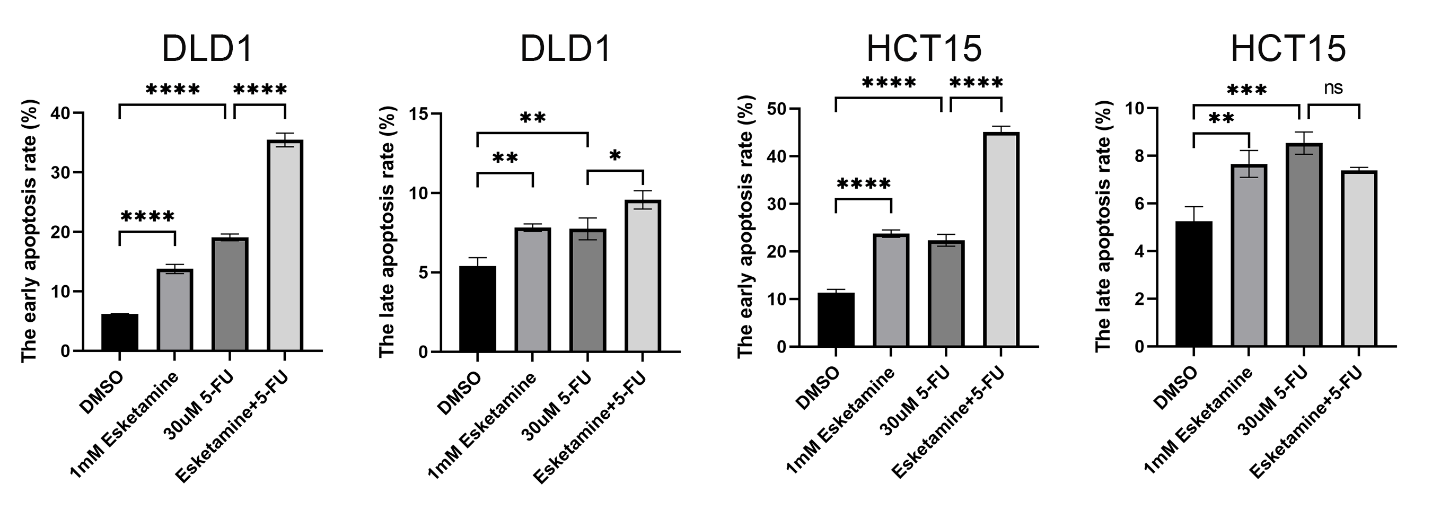
**

**Supplementary Figure S2. Quantitative analysis of early and late apoptosis in DLD1 and HCT15 cells.** Bar graphs showing the percentages of early apoptotic (Annexin V+/PI–), late apoptotic (Annexin V+/PI+) cells following treatment with esketamine (1 mM), 5-FU (30 μM), or their combination for 48 h. Data are presented as mean ± SD (n = 3 independent experiments). **P* < 0.05, ***P* < 0.01, ****P* < 0.001, *****P* < 0.0001; ns, not significant.

**
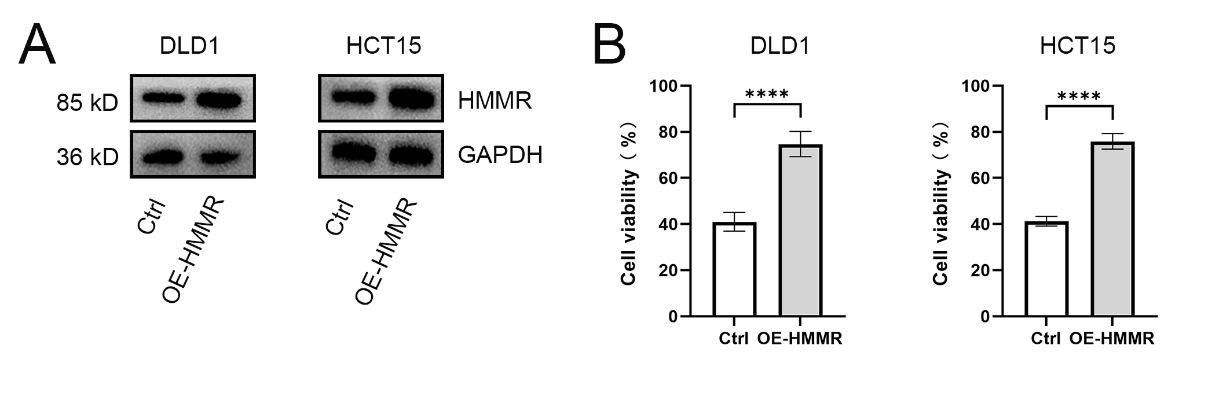
**

**Supplementary Figure S3. HMMR overexpression attenuates the anti-viability effect of the esketamine and 5-FU combination.** (A) Western blot validation of HMMR overexpression in stable HMMR-OE DLD1 and HCT15 cell lines. (B) Cell viability assessed by CCK-8 assay in control and HMMR-OE cells treated with esketamine (1 mM) and 5-FU (30 μM) for 48 h. Data are presented as mean ± SD (n = 3 independent experiments). *****P* < 0.0001 vs. respective control group.
